# Zirconium Surface Treatment via Chemical Etching

**DOI:** 10.3390/ma16237404

**Published:** 2023-11-28

**Authors:** Przemysław Gołasz, Agata Kołkowska, Rafał Zieliński, Wojciech Simka

**Affiliations:** 1 Department of Inorganic Chemistry, Analytical Chemistry and Electrochemistry, Faculty of Chemistry, Silesian University of Technology, 44-100 Gliwice, Poland; pg292060@student.polsl.pl (P.G.); agatkol653@student.polsl.pl (A.K.); 2 Chemistry Students Research Society, Faculty of Chemistry, Silesian University of Technology, 44-100 Gliwice, Poland; 3 Stomatologia na Księżym Młynie, 90-365 Łódz, Poland

**Keywords:** zirconium, dental implant, surface treatment, etching

## Abstract

The increased demand for implants that do not pose a threat to patients diagnosed using high-resolution magnetic resonance imaging and concerns arising from titanium allergies require the development of alternative implant materials. One promising concept is a use of zirconium as corrosion-resistant, nontoxic material that is lower in magnetic susceptibility. To achieve this, safe and efficient surface treatment methods of zirconium metal have to be developed. In this study, zirconium samples were treated with fluoride-free and fluoride-containing etching mixtures to determine their effect on the surface of Zr. SEM images were taken to investigate the preliminary effects of the etchants. Then, a second set of experiments was carried out using mixtures of HF-H_2_SO_4_ and ammonium persulfate–fluoride salts, as they gave the most promising results in the first trial. SEM images were taken and measurements on roughness, wettability, and atomic composition were made. The results showed an even zirconium surface in APS-fluoride salts, along with the formation of pits (1–3 μm) similar to those found in commercially available implants. There was no significant increase in the roughness of the treated samples. The addition of NO_3_^−^ ions in the form of KNO_3_ speeded up etching and promoted pit formation. The HF-H_2_SO_4_ mixture was found to give unsatisfying results, as the surface was too rough and the formed pits were too large. It was concluded that etching zirconium in ammonium persulfate and fluoride salts is a promising area of research for the preparation of zirconium implants; however, further research has to be carried out on sandblasted samples.

## 1. Introduction

The field of implantology has undergone remarkable advances in recent years, offering innovative solutions to restore both function and aesthetics to surgical patients. Implant materials play an essential role in the success of these procedures, with a wide range of options available to clinicians. A frequently used and well-studied material is titanium [1,2,3,4]. It is often alloyed with other metals to achieve better mechanical strength, ductility, corrosion resistance, or osseointegration. The most common titanium alloy used in implantology is Ti6Al4V [3,4], but other metals such as molybdenum, zirconium, niobium, or tantalum can also be used [3,5]. The titanium–zirconium alloy (TiZr1317) containing 13–17% zirconium developed by Straumann shows a very similar topography to the titanium implant with a similar biocompatibility with the additional benefit of being 50% stronger than pure titanium [5]. Additionally, studies and analysis were carried out on titanium–zirconium alloys, which confirmed zirconium biocompatibility and the potential of titanium–zirconium alloys’ corrosion resistance and mechanical durability [6,7]. However, with a huge number of articles describing etching methods and their effect on the osseointegration of titanium and titanium-based alloys [7,8,9] and zirconia (zirconium dioxide used as dental implant material) [10,11,12,13], there is little to no literature describing those processes and properties for zirconium-metal-based alloys.

Zirconium and titanium have very similar mechanical properties to bones (Young’s modulus and hardness), and also good corrosion resistance in the body environment [14]. Various methods of titanium surface treatments have been successfully developed to increase the optimal level of osseointegration, which is mandatory for the stability and durability of the bone-to-implant connection [3,15,16]. However, there is no researched method for the preparation of zirconium metal to provide this osseointegration. Osseointegration is the process of fusion of the implant with the bone, without connective tissue between them. In order to form a connection with the implant, there must be characteristic micro- and nanostructures that would allow osteoblasts and supporting connective tissue to migrate and mature [17]. One of the most prominent ways to ensure osseointegration for titanium is preparation through the SLA treatment (SLA stands for sandblasted, large-grit, acid-etched) [18]. Sandblasting creates an uneven, rough surface, and acid etching further promotes the formation of cavities and bogs with sharp edges and a low level of surface organization [19,20]. An unorganized and rough surface is preferred by the human osteoblast cell [21]. A similar treatment should be researched for zirconium in order to increase bone-to-implant-surface contact.

Zirconium has been found to have a low magnetic susceptibility of 1.32 × 10^−6^ cm^3^g^−1^. It has recently been discovered that alloying Zr with other metals such as Nb and Sn to form Zr-xNb-4Sn can result in a reduction in magnetic susceptibility and Young’s modulus, making it a potential implant material [22,23]. This research is crucial in meeting the demand for magnetic resonance imaging using a strong electromagnetic field >3.0 T to obtain more accurate images with a higher resolution. This tendency in medical imaging increases the demand to search for suitable materials. For example, titanium alloys or other metals used in implantology, such as stainless steel, have a relatively high magnetic susceptibility (3.2 × 10^−6^ cm^3^g^−1^ for titanium alloys and 93.1 × 10^−6^ cm^3^g^−1^ for 316 stainless steel). Magnetic susceptibility is a factor that measures how much a material becomes magnetized when subjected to an external magnetic field. Stainless steel and titanium alloys magnetize above 3.0 T. That process may lead to the heating, shifting, and detachment of implants, as well as distort imaging and create artifacts on the images [22,24].

Another concern associated with the use of titanium as an implant material is the risk of allergic reactions in patients. As is often overlooked by medical professionals, titanium implants can cause dermal allergic reactions and the inflammation of the tissue around the implant in the postoperative period—the effects are not well-understood [25,26]. Such effects were not found yet in clinical practice for zirconium usage, and no other concerns involving metallic zirconium have been described.

Zirconium is known for its outstanding resistance to chemical and corrosion [27,28]. It is an active metal that reacts with atmospheric oxygen, water, and acids, but the exposed layer of metallic zirconium is immediately covered with a layer of amphoteric zirconium oxide. Zirconium ions have a high affinity for oxide anions [29], creating a layer that is insoluble by most etching substances.

The current state of research on zircon surface preparation only describes its dissolution in hydrofluoric acid. That is because fluoride ions can form soluble complex ions and are stable in water. According to the research, the rate of zirconium dissolution can be increased by the addition of strong acids, such as nitric, sulfuric(VI), and hydrochloric acids [30]. However, those mixtures are known to be notoriously dangerous and corrosive, making working with them dangerous, especially on a larger industrial scale. There are no other researched methods known to the authors that successfully etched zirconium metal.

To achieve the acceptable osseointegration of zirconium implants, it is crucial to create a surface of suitable roughness and topography. Methods of zirconium etching have to be developed to safely and effectively manufacture zirconium implants on a large scale safely and effectively. Ideally, a way of etching zirconium and its alloys without the need for fluoride usage has to be developed to ensure the safety of the environment and workers; however, a significant decrease of the acidity and an increase of the stability of etching mixtures will be sufficient.

In this paper, pure zirconium (Zr 99.9%) was etched in various mixtures. The experiments focused only on developing an etching mixture that creates a similar unorganized surface with the promotion of the formation of pits and pores. Because Zr is in the same group of the periodic table as Ti, it was proposed that zirconium etching can be performed using the fluoride-free etchants used in Ti etching, such as sulfuric(VI) and oxalic acids [31,32]. Furthermore, citric acid and its mixture with oxalic acid was used as a possible simple yet effective complexing ion. Despite the resistance of zirconium to alkaline conditions, a phosphate-containing mixture with NaOH and Na_3_PO_4_ is used to investigate its effects on the zirconium surface. Those mixtures were expected to etch zirconium in approximately one hour. In addition, mixtures containing fluoride ions were investigated. As fluoride ions are considered mandatory for dissolving the zirconium oxide layer and an acid environment increases the rate of the oxidation of zirconium metal [33,34,35], mixtures containing fluoride salt (NH_4_F and NaF) were prepared with the addition of a mildly acidic oxidizing agent ((NH_4_)_2_S_2_O_8_). The mixture was expected to etch Zr slightly at a slower and more controlled manner, along with being more stable and producing less vapor in the process compared to mixtures of HF with strong acid. Furthermore, the effect of more conventional mixtures of H_2_SO_4_ and HNO_3_ with HF was investigated to compare the results with the alternative methods. The surface topography, roughness, and wettability of the etched surfaces were examined. Additionally, the chemical composition was analyzed using energy-dispersive X-ray spectroscopy.

## 2. Materials and Methods

Discs made of pure zirconium (3 mm thick, with diameter of 9 mm) were used (Zr 99.9% BIMO Metals, Wrocław, Poland). Each disc was polished using abrasive SiC paper of up to 1000 grit. The samples were rinsed in distilled water and degreased in isopropanol in an ultrasonic bath (7 min), and then in distilled water (7 min). The samples were divided into two groups—A (fluoride-free trial group) and B (fluoride-containing trial group). Group A samples were prepared according to the current state of knowledge for etching titanium samples without the use of fluorides, as there is no literature describing dissolution of zirconium without fluoride ions. Samples from group B, etched in fluorine-containing solutions, require a much shorter etching time, so they were prepared accordingly [31,32,33,34,36].

The zirconium discs from group A were etched in 90 °C plastic cups in ultrasonic baths at 90 °C. Discs were placed on the bottom of the cups and left with ultrasounds on for 45, 60, and 75 min. The etched samples were washed in distilled water for 2 min and ultrasonically cleaned in distilled water for 15 min, followed by drying at room temperature for 2 h. Discs from group B were etched in room-temperature mixtures contained in plastic beakers. The discs were held with titanium tweezers and constantly stirred during etching. Next, the etched samples were then washed in distilled water for 2 min and ultrasonically cleaned in distilled water for 15 min, followed by drying at room temperature for 2 h. Table 1 shows labeling of etching mixtures and their compositions. To determine preliminary results of experiments, scanning electron microscopy (SEM) was used (Hitachi TM3000, Tokyo, Japan). The applied voltage was 15 kV. SEM sample images were compared to images of an unetched sample of zirconium. Samples of groups A and B that showed significant differences in surface topography compared to unetched zirconium were noted.

After analyzing group A and B samples, based on the obtained results, main etching mixtures were developed. Samples etched in main group mixtures (group M) were etched in room-temperature mixtures contained in plastic beakers. The discs were held with titanium tweezers and constantly stirred during etching. The etched samples were washed in distilled water for 2 min and ultrasonically cleaned in distilled water for 15 min, followed by drying at room temperature for 2 h. Table 2 shows the labeling of the main etching mixtures and provides information about their composition and etching times of samples in given mixtures. The discs were investigated using SEM equipped with energy-dispersive X-ray spectroscopy to determine the elemental composition of the surface achieved (Phenom Pro-X, Thermo Fisher Scientific Inc., Waltham, MA, USA). The applied voltage was 15 kV. Furthermore, the mean arithmetic roughness value Ra and mean roughness depth Rz were determined (Mitutoyo S-301J, Kanagawa, Japan) using six measurements on two independent samples. Additionally, the Sa values were determined using a Phenom Pro-X microscope. The wettability of the samples was determined using a goniometer (DataPhysics, OCA 15EC, Filderstadt, Germany) by measuring contact angles. To measure the contact angle as a function of time, a 0.2 µL drop of deionized water was dropped on the surfaces and the contact angle was measured. Additionally, the initial contact angle was determined using 0.2 µL of water dropped on the surface of the sample and immediately measuring the angle. The results are mean values derived from six measurements from two independent samples.

## 3. Results

### 3.1. Preliminary Investigations

**Figure 1:**
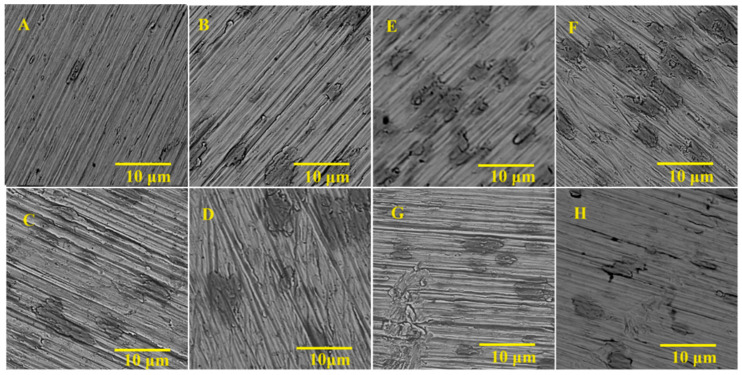
The SEM (scanning electron microscope) images of A group zirconium samples: (**A**) machined, (**B**) A-CIT30 etched for 75 min, (**C**) A-SULF70 etched for 60 min, (**D**) A-SULF70 etched for 75 min, (**E**) A-OXA7,5 etched for 75 min, (**F**) A-OXA20 etched for 75 min, (**G**) A-PHOS10 etched for 75 min, and (**H**) A-CITOXA15-10 etched for 60 min.

**Figure 2:**
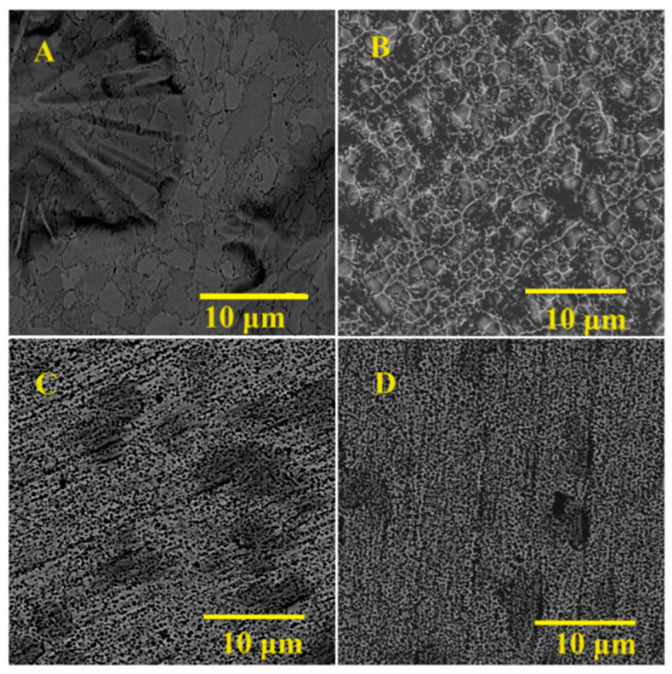
The SEM (scanning electron microscope) images of B group samples: (**A**) B-HNO_3_ etched for 30 s; (**B**) B-NaF2 etched for 45 s; (**C**) B-NaF3 etched for 45 s; and (**D**) B-NaF4 etched for 45 s.

Zirconium samples etched in group A mixtures (fluoride-free trial) did not show significant differences compared to unetched zirconium samples. Figure 1 presents SEM images of etched surfaces in fluoride-free etchants and a machined sample. There are visible grooves and protruding crystals of zirconium metal created after mechanical processing. The overall surface profile remained unchanged for all group A samples, which changed the course of the study from the development of fluoride-free zirconium etchants to the development of a possibly safe and strong acid-free etching mixture. Samples etched in group B (fluoride-containing trial) etchants showed changes in surface topography and overall appearance. Figure 2 shows SEM images of zirconium samples treated with group B etchants. The surface structure of the B-NaF2-treated sample resembled implant surfaces [3,6,7,10] with an unorganized structure with pits and cavities. The B-NaF3 and B-NaF4 samples had smaller pits and pores. Their surface showed dark spots that are also visible in group A etched samples, indicating incomplete surface etching. The sample treated with B-HNO_3_ showed no visible porosity, but the surface was lustrous, shiny, and smooth. Taking into account all the data collected, it was decided that the main trials would be carried out using the etchants listed in Table 2.

### 3.2. Main Samples’ Surface Morphology

**Figure 3:**
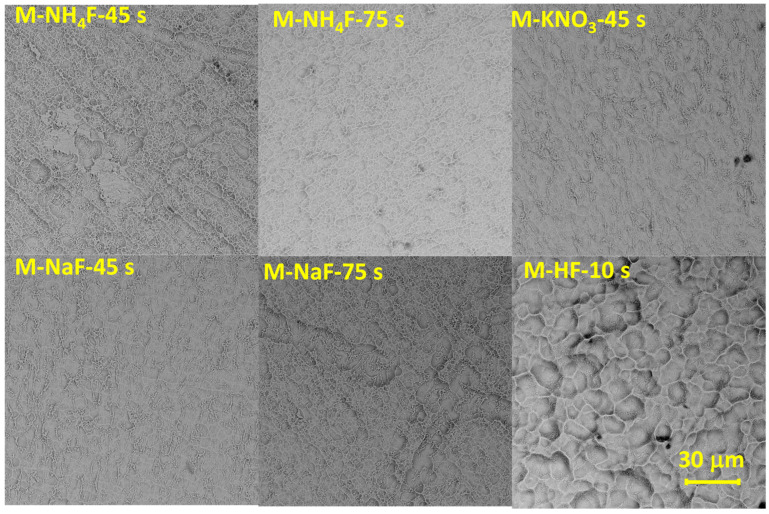
The SEM images of samples etched in main group etchants: NH_4_F etched for 45 s; M-NH4F etched for 75 s; M-KNO_3_ etched for 45 s; M-NaF etched for 45 s; M-NaF etched for 75 s; and M-HF etched for 10 s.

Samples treated with the main-group etchants were investigated under SEM (Phenom Pro-X). The M-HF-etched disc had wide (around 10 μm) pores with smoothness. Samples etched in fluoride salts (M-NH_4_F, M-NaF, and M-KNO_3_) developed pits of smaller diameter and lower height amplitudes than the M-HF-etched sample. There are clusters of small, unorganized pits (diameter > 1 μm) on M-NH_4_F and M-NaF etched for 75 s, which are irregular and surrounded by uniform and relatively flat surfaces. The M-NH_4_F and M-NaF etched for 45 s samples showed visible grinding marks and a porous surface (pores of 1–3 μm diameter). The surface of M-KNO_3_ was the most unorganized with a lot of holes (approximately 1–3 μm in diameter) with no visible grinding marks. Figure 3 shows the M group SEM images.

### 3.3. Main Samples’ EDX

**Figure 4:**
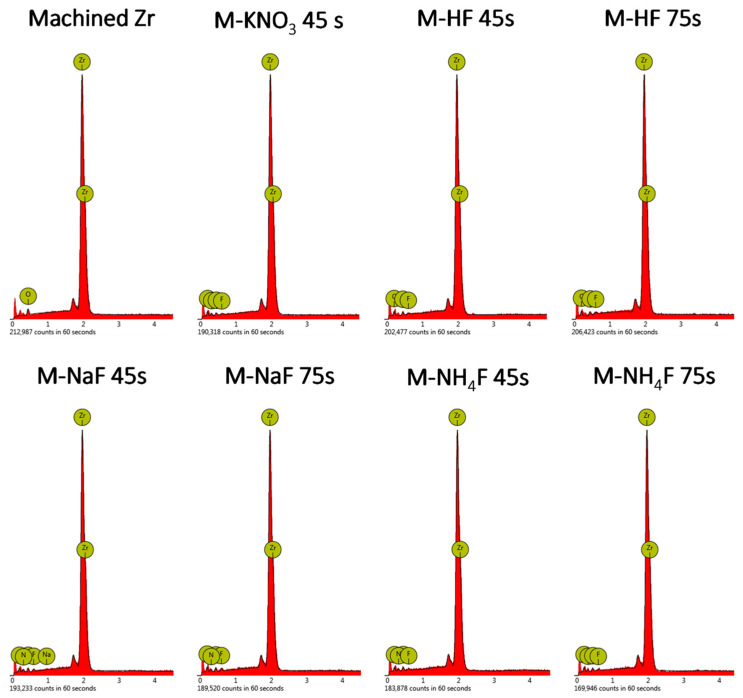
The SEM images and EDX spectra of main group samples.

EDX analysis confirmed the presence of elements such as Zr, O, N, C, F, and trace amounts of Na. The surface of samples etched in nitrogen-containing mixtures (M-NH_4_F, M-NaF, and M-KNO_3_) exhibits a composition very similar to that of the nitrogen detected. The presence is caused by the absorption of CO_2_ from air and water on the surface of the samples. Figure 4 shows the SEM images of the surfaces analyzed and the EDX (energy-dispersive X-ray spectrometer) mass concentration spectra of the main group samples. Table 3 shows the atomic composition of the samples (%) indicated by the semiquantitative EDX analysis of the treated samples.

### 3.4. Main Samples’ Surface Roughness

The arithmetic mean roughness value for a trace (R_a_), the mean roughness depth for a trace (Rz), and the arithmetic mean roughness value for an area (S_a_) were determined for the machined zirconium sample from the main group samples. The data were collected in Table 4. The most significant difference in roughness from the untreated sample was achieved in the M-HF sample. The increase in etching time increased the roughness of the surface in M-NaF and M-HF, but decreased it in the M-NH_4_F sample.

### 3.5. Main Samples’ Surface Wettability

**Figure 5:**
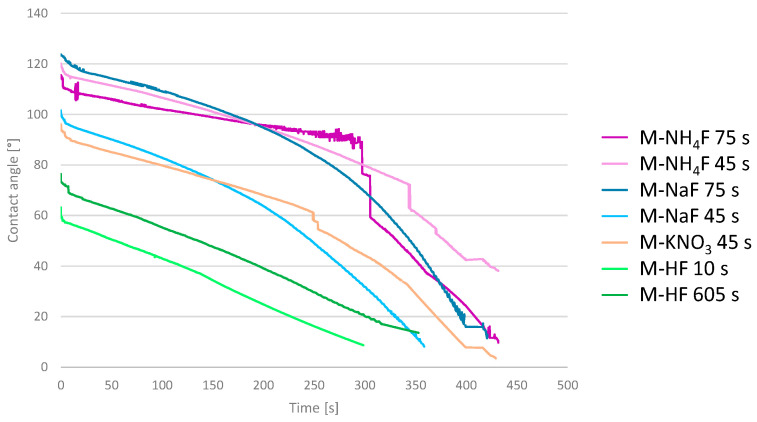
The contact angle of the main group samples is shown as a function of time.

Wettability was determined using contact angle measurements. The static contact angles are collected in Table 4. In addition, the dynamic contact angle was determined as a change in the contact angle over time. Those values were plotted against the graph and are shown in Figure 5. The shortest time for a water drop to spread on the surface was measured for M-HF etched for 10 s and was equal to 300 s. Both M-HF-etched samples showed this same, simple, linear decay of contact angle. M-KNO_3_, M-NH_4_F 75 s, and M-NaF 75 s showed an approximate contact angle decay equal to 430 s. The 45 s M-NH_4_F contact angle change reached 38.8 at 430 s, and, after that, the measurement was terminated. Samples etched in M-NaF, M-NH_4_F, and M-KNO_3_ had the same logarithmically shaped curve of contact angle decay, increasing the decline rate with time. It can be seen that samples etched in acidic mixtures of M-HF were much more hydrophilic than the ones etched in mixtures excluding the use of acids.

## 4. Discussion

Zirconium metal, as a potential implant material, exhibits some unique properties, making it an interesting material for making bone implants that are safe when used during MRI diagnosis [22,24,25,26]. One of the challenges is to find a safe and suitable method of surface treatment to ensure the osseointegration of the implant with the bone. The results of the surface treatment with the use of various fluoride-free and fluoride-containing mixtures on the zirconium metal are shown in this paper. Although all proposed fluoride-containing mixtures were able to successfully etch the zirconium surface, a solution of 48% HNO_3_ and 10% HF was found to give unsatisfying results. The surface of zirconium was shiny and lustrous, with small grains and metal crystals on the surface. The SEM images showed no pores, pits, or cavities, which are essential for improving the osseointegration of the bone to the implant [17,21]. Mixtures containing NaF in concentrations of 2.10%, 3.15%, and 4.20%, and 10% APS were investigated. Etching in A-NaF2 resulted in the formation of pits with a diameter of around 1–2 μm and a uniform surface. An increase in the sodium fluoride concentration to 3.15% and 4.20% of NaF resulted in the formation of more smaller diameter pits (>1 μm) on the surface of the samples. Additionally, the dark marks visible on the unetched zirconium were more visible, along with the grinding marks, especially on the surface of A-NaF3. It was concluded that the usage of concentrations of fluoride ions similar to those in A-NaF2 should be used in further tests, as it provided the most satisfying and interesting result. Etching zirconium in group B etchants showed no change in the surface structure. In all samples, there are visible grinding marks, dark spots, and crystals on the surface of the zirconium; therefore, further experiments with fluoride-free etchants were terminated.

The next step was to develop a formula for possibly the safest mixture that can create an unorganized structure capable of osseointegration. Further tests using more advanced analytical methods were conducted on samples etched in M-NaF and M-NH_4_F, which showed very similar results to each other. The discs etched in those solutions for 45 s exhibit porous but uneven surfaces with still-visible grinding marks and diameters of around 1–3 μm. Samples etched in M-NaF and M-NH_4_F for 75 s have no visible surface deformations from mechanical processing; however, their surface porosity is significantly decreased compared to samples etched for 45 s. The sample surface of M-KNO_3_ 45 s is more even etched than discs etched in solutions of M-NaF 45 s and M-NH_4_F 45 s, without residues after mechanical processing. It has visible holes with a diameter of around 1–3 μm. It is possible that NO_3_^−^ ions in slightly acidic conditions increase the rate of dissolution of zirconium while maintaining a porous structure [33,34,35]. Pits of similar diameter [19] are often formed during the SLA treatment of titanium implants; however, their depth and morphology are quite different, as previously mentioned, due to the additional sandblasting treatment. Samples etched in M-HF for 10 and 60 s have larger pits (around 3–6 μm in diameter) than samples treated with fluoride salt-based etchants. The surface of those samples was uneven, with bulges and smooth, deep pores.

The roughness of the formed surfaces was determined by measuring Ra, Rz, and S_a_, as these are among the factors influencing bone-to-implant integration. Those remained relatively the same compared to the untreated samples in discs etched in M-NH_4_F, M-NaF, and M-KNO_3_. However, Figure 2 and Figure 3 show that elongating the etching time changes the topography of the surface of the treated samples (Figure 2—comparing A to B and Figure 3—D to E) without significant changes in the Ra and Rz compared to the untreated disc. Treating zirconium in an M-HF solution greatly increased its surface values of R_a_, Rz, and S_a_. Etching the sample in M-HF for 60 s increased the Ra value by almost eight times, and the Rz value by almost six times, which is much too high for implantology use, as osseointegration implants that are commercially used achieve Ra values of around 0.4 to 1.0 μm [37].

The wettability of implants is quite an undiscovered area, as different implant surfaces exhibit very different wettability. The SLA surfaces of Ti6Al4V range from 117 to 150 contact angles, while, this same alloy, but SLActive-surface-treated, ranges from 0 to 5. However, most implant surfaces oscillate around 100 CA [38]. The contact angle greatly varies between different implant surfaces; it is a non-obvious variable and its impact on osseointegration is not well-studied. However, the CA is still a great indicator of surface hydrophilicity, which, although unclear, plays a significant role in bone-to-implant integration. In this study, surfaces treated with M-NaF showed the highest static contact angles. M-NH_4_F and M-KNO_3_ showed very similar results in the static contact angle. All of those samples had a quite similar change of contact angle over time. These results indicate an increase in the hydrophobic properties of the samples compared to those of machined zirconium. The graphs showed a smooth, logarithmic decay, with initial values higher than those of machined zirconium. Treating the samples with M-HF resulted in a great decrease in static contact angles; in addition, their dynamic contact angles showed a fast linear decay over time. These results are caused by the larger size of the pits on M-HF-treated surfaces compared to the rest of the samples, which makes it easier for water to penetrate.

An EDX analysis showed the presence of oxygen and carbon in all of the etched samples. The most likely oxygen content is caused by the formation of various crystal structures of zirconia (zirconium(IV) oxide) on the implant surface, as it is the main component of the zirconium passivation layer [30,39]. Carbon on the surface of the samples probably originates from atmospheric or equipment sources, such as carbon dioxide, carbon tape used in SEM imaging, and organic impurities. It is worth noting that samples etched in nitrogen-containing etchants (M-NaF, M-NH_4_F, and M-KNO_3_) have nitrogen introduced into the surface of the samples. It is yet unknown in what form the nitrogen is present; however, based on the etching mixtures, it is possible for ammonium salts and complexes to form. In all samples, there is a relatively small content of fluorine present, which can hopefully be decreased by post-etching surface treatment.

## 5. Conclusions

This study shows that the treatment of zirconium metals with solutions containing ammonium persulfate and fluoride salts may be a promising area of research for the preparation of zirconium implants. The use of such mixtures developed less organized surfaces with visible pits compared to the use of HF-HNO_3_ and HF-H_2_SO_4_ etching mixtures. The presence of NO_3_^−^ ions promotes an even dissolution of the zirconium surface while promoting the formation of evenly distributed pits. This treatment did not significantly alter the roughness of the surfaces, as the values of R_a_, R_z_, and Sa remained relatively similar to those of machined zirconium. The surfaces were found to be more hydrophobic than machined zirconium, with an increase in the contact angle of about 10 to 20 degrees.

## Figures and Tables

**Table 1:** Labeling, composition, and etching times of preliminary etchants.

	Label	Composition [*w*/*w*]	Etching Time
Group A	A-OXA7.5	7.5% oxalic acid	75 min
	A-OXA10	10% oxalic acid	75 min
	A-OXA15	15% oxalic acid	75 min
	A-OXA20	20% oxalic acid	45, 60, 75 min
	A-CIT30	30% citric acid	45, 60, 75 min
	A-CITOXA15-10	15% citric acid; 10% oxalic acid	75 min
	A-PHOS10	10% Na_3_PO_4_; 10% NaOH	45, 60, 75 min
	A-SULF70	70% H_2_SO_4_	45, 60, 75 min
Group B	B-NaF2	10.0% (NH_4_)_2_S_2_O_8_; 2.10% NaF	45, 60, 75 s
	B-NaF3	10.0% (NH_4_)_2_S_2_O_8_; 3.15% NaF	45, 60, 75 s
	B-NaF4	10.0% (NH_4_)_2_S_2_O_8_; 4.20% NaF	45, 60, 75 s
	B-HNO_3_	48%HNO_3_; 10% HF	20, 30 s

**Table 2:** Labeling, composition, and etching times of main etchants.

	Composition [*w*/*w*]	Etching Times
M-NH_4_F	10.0% (NH_4_)_2_S_2_O_8_; 1.85% NH_4_F	45, 75 s
M-KNO_3_	10.0% (NH_4_)_2_S_2_O_8_; 1.85%NH_4_F; 3% KNO_3_	45 s
M-HF	17.4% H_2_SO_4_; 1% HF	10, 60 s
M-NaF	10.0% (NH_4_)_2_S_2_O_8_; 2.10% NaF	45, 75 s

**Table 3:** The semi-quantitative EDX analysis of treated samples, composition in atomic composition (%).

	Etching Time, s	Zr	C *	N *	O *	F *
Machined Zr	—	92	—	—	8	—
M-NaF	45	41	28	18	10	3
	75	38	31	17	10	3
M-NH_4_F	45	43	27	20	9	2
	75	39	31	22	7	1
M-KNO_3_	45	41	33	20	6	1
M-HF	10	50	39	-	10	1
	60	47	41	-	10	2

* The values must be regarded as only informative.

**Table 4:** Surface roughness and initial contact angle of water droplet on surface of machined Zr and main group samples.

	Etching Time, s	R_a_, μm	R_z_, μm	S_a_, μm	Average Contact Angle, °
Machined Zr	—	0.43	3.94	0.95	93.8
M-NaF	45	0.33	2.80	0,91	112.9
	75	0.46	3.29	1.49	116.1
M-NH_4_F	45	0.42	3.34	1.27	107.1
	75	0.34	2.75	0.87	106.2
M-KNO_3_	45	0.42	3.37	1.16	103.3
M-HF	10	0.83	5.94	3.05	59.0
	60	3.28	18.32	3.21	55.4

## Data Availability

The data are available upon request.
